# Assessment of copy number variations in 120 patients with Poland syndrome

**DOI:** 10.1186/s12881-016-0351-x

**Published:** 2016-11-25

**Authors:** Carlotta Maria Vaccari, Elisa Tassano, Michele Torre, Stefania Gimelli, Maria Teresa Divizia, Maria Victoria Romanini, Simone Bossi, Ilaria Musante, Maura Valle, Filippo Senes, Nunzio Catena, Maria Francesca Bedeschi, Anwar Baban, Maria Grazia Calevo, Massimo Acquaviva, Margherita Lerone, Roberto Ravazzolo, Aldamaria Puliti

**Affiliations:** 1 Department of Neurosciences, Rehabilitation, Ophthalmology, Genetics, Maternal and Child Health (DiNOGMI), University of Genoa, Genoa, Italy; 2 Medical Genetics Unit, Istituto Giannina Gaslini, Genoa, Italy; 3 Pediatric Surgery Unit and Airway Team, Istituto Giannina Gaslini, Genoa, Italy; 4 Department of Genetic Medicine and Development, Geneva University Medical School, Geneva, Switzerland; 5 Plastic and Reconstructive Surgery, Pediatric Surgery Unit, Istituto Giannina Gaslini, Genoa, Italy; 6 Radiology Unit, Istituto Giannina Gaslini, Genoa, Italy; 7 Reconstructive Microsurgery and Hand Surgery Unit, Istituto Giannina Gaslini, Genoa, Italy; 8 Medical Genetics Unit, Fondazione IRCCS Ca’ Granda Ospedale Maggiore Policlinico, Milan, Italy; 9 U.O.S.D. Epidemiology and Biostatistics, Istituto Giannina Gaslini, Genoa, Italy; 10 Medical Genetics Unit, Istituto Giannina Gaslini, via G. Gaslini 5, 16148 Genoa, Italy; 11 Present Address: Department of Pediatric Cardiology and Cardiac Surgery, Bambino Gesù Children’s Hospital, Rome, Italy

**Keywords:** Array comparative genomic hybridization, Congenital abnormalities, DNA copy number variation, Limb anomalies, Musculoskeletal diseases, Pectoralis muscles, Poland syndrome, Chromosome deletion, Chromosome duplication

## Abstract

**Background:**

Poland Syndrome (PS) is a rare congenital disorder presenting with agenesis/hypoplasia of the pectoralis major muscle variably associated with thoracic and/or upper limb anomalies. Most cases are sporadic, but familial recurrence, with different inheritance patterns, has been observed. The genetic etiology of PS remains unknown. Karyotyping and array-comparative genomic hybridization (CGH) analyses can identify genomic imbalances that can clarify the genetic etiology of congenital and neurodevelopmental disorders. We previously reported a chromosome 11 deletion in twin girls with pectoralis muscle hypoplasia and skeletal anomalies, and a chromosome six deletion in a patient presenting a complex phenotype that included pectoralis muscle hypoplasia. However, the contribution of genomic imbalances to PS remains largely unknown.

**Methods:**

To investigate the prevalence of chromosomal imbalances in PS, standard cytogenetic and array-CGH analyses were performed in 120 PS patients.

**Results:**

Following the application of stringent filter criteria, 14 rare copy number variations (CNVs) were identified in 14 PS patients in different regions outside known common copy number variations: seven genomic duplications and seven genomic deletions, enclosing the two previously reported PS associated chromosomal deletions. These CNVs ranged from 0.04 to 4.71 Mb in size. Bioinformatic analysis of array-CGH data indicated gene enrichment in pathways involved in cell-cell adhesion, DNA binding and apoptosis processes. The analysis also provided a number of candidate genes possibly causing the developmental defects observed in PS patients, among others *REV3L,* a gene coding for an error-prone DNA polymerase previously associated with Möbius Syndrome with variable phenotypes including pectoralis muscle agenesis.

**Conclusions:**

A number of rare CNVs were identified in PS patients, and these involve genes that represent candidates for further evaluation. Rare inherited CNVs may contribute to, or represent risk factors of PS in a multifactorial mode of inheritance.

**Electronic supplementary material:**

The online version of this article (doi:10.1186/s12881-016-0351-x) contains supplementary material, which is available to authorized users.

## Background

Poland Syndrome (PS, MIM173800) is a congenital disorder of the pectoralis major muscle. PS patients present with pectoralis muscle agenesis/hypoplasia, more frequently on the right side. PS can be associated with a variable degree of ipsilateral thoracic and/or upper limb anomalies [1, 2]. Incidence of PS has been reported between 1/20,000 and 1/30,000 births with a higher prevalence in males [3, 4]. Today, PS etiopathogenesis is still unknown. One of the most common assumptions is that isolated pectoralis major muscle defects are included in the spectrum of anomalies postulated to result from disruption of blood supply in the embryonic subclavian and vertebral arteries [5, 6]. Alternatively, PS may be due to the involvement of genes regulating embryonic development of pectoral girdle [7]. Familial recurrence was observed in about 10% of cases with different inheritance patterns including autosomal dominant with incomplete penetrance, autosomal recessive, and X-linked [1]. The presence of different genes whose mutations may account for clinical differences among subgroups of patients and for the different inheritance patterns observed could be hypothesized. Two recently reported cases of de novo deletions contribute to support the genetic origin of PS and suggests the involvement of the deleted regions in PS pathogenesis. A deletion of chromosome 11q12.3 in monozygotic twins both affected by PS [8] and a large deletion of chromosome 6q21-q22.1 in a patient with a complex phenotype mainly characterized by mental disability and PS [9]. Genomic imbalances and copy number variants (CNV) represent a main source of genetic variation in humans and contribute to different congenital and neurodevelopmental defects [10–14]. However, the contribution of genomic imbalances to a broader number of PS patients has not been systematically studied. In this study, we performed karyotyping and array-CGH analysis of a large cohort of PS patients to discover novel chromosomal regions associated with this condition.

## Methods

### Patients

The present cohort comprises 120 patients with pectoralis muscle agenesis/hypoplasia, either isolated or with associated anomalies (clinical data are summarized in Table 1). Control population (in-house controls) comprises 200 patients affected by various disorders (mainly intellectual disabilities) admitted to the Medical Genetics Unit, Gaslini Institute, Genoa, Italy, between January 2008 and December 2015, and their healthy parents for a total of 600 control individuals with no clinically evident pectoralis muscle abnormalities nor other features usually reported to be associated with PS.

**Table 1:** Summary of clinical data of all 120 patients

Clinical feature	Total patients 120
Gender	
Females	44
Males	76
Pectoralis major muscle features	
Agenesis	51
Hypoplasia	69
Right-sided	71
Males	44
Females	27
Left-sided	49
Males	33
Females	16
Isolated form	42
With associated anomalies	78
Associated anomalies	
Pectus excavatum/carinatum	21
Rib anomalies	10
Clavicle defects	1
Vertebral defects	1
Craniosynostosis	1
Upper limb anomalies	57
Classic brachysyndactyly	24
Hypoplasia of upper limb and/or hand	28
Cerebellar malformations and intellectual disabilities	1
Poland-Möbius syndrome	1

All patients were evaluated by a multidisciplinary team enclosing surgeons, radiologists, orthopaedic surgeons, and clinical geneticists to get a complete evaluation of the patient phenotype. The presence of additional anomalies as dysmorphic signs, and the presence of associated syndromic features were carefully evaluated by a team of clinical geneticists with experience in dysmorphology. PS specific features and the possible presence of additional anomalies were investigated in details by physical examination, followed by radiological/ultrasound examination.

### Karyotyping and array-CGH analyses

Chromosome analysis was carried out on GTG-banded chromosomes at a resolution of 550 bands. Array-CGH analysis was performed using a genomic oligonucleotide-array with 13 kb (AMADID 022060) or 22 kb (AMADID 014698) (Human Genome Microarray Chip; Agilent Technologies, Palo Alto, CA, USA). Array data were analyzed using the Agilent Genomic Workbench Lite Edition Software 6.5.0.18. Aberration segments were reviewed using GRCh37 hg19 of UCSC Genome Browser (http://genome.ucsc.edu/index.html). We annotated all detected copy number variations (CNVs) and CNV-encompassed genes across public databases: Genomic Variants Database (DGV) (http://dgv.tcag.ca/dgv/app/home), DECIPHER (https://decipher.sanger.ac.uk/), Clinical Genome Resource (ClinGen) and ISCA (http://www.clinicalgenome.org), OMIM (http://www.omim.org), PubMed (http://www.ncbi.nlm.nih.gov/pubmed), and databases of mouse (MGD)(http://www.informatics.jax.org) and zebrafish (ZFIN) (ZFIN, http://zfin.org) models.

### CNV annotation

All detected CNVs were tested for inheritance by hybridization of the parental DNA with the same array platform. A CNV was classified as unreported if it differed from those already reported for involving one more gene and/or if it differed by at least 100 kb on either side or by a total of 100 kb on both sides. To assess the clinical significance of the detected CNVs, we followed the recommended steps from Miller and coll. [15]. All imbalances classified as benign in the ClinGen and/or found in our internal database of healthy individuals were considered to be benign and excluded from further analysis. The remaining CNVs were classified into groups. Group I contains genomic imbalances classified as being variants of uncertain clinical significance (VOUS) because of their unclear possible pathogenicity. CNVs were further classified as VOUS likely pathogenic, if including genes with a possible correlation to the phenotype, or simply VOUS, those CNVs for which clinical interpretation remains uncertain. Group II contains pathogenic CNVs overlapping critical regions of known microdeletions or microduplications and/or involving genes already described as causing a phenotype. These CNVs are found in the publicly available DECIPHER (https://decipher.sanger.ac.uk/) and ISCA (www.clinicalgenome.org) databases and in published literature. Only VOUS, and VOUS likely pathogenic variants were further investigated.

### Real-Time genomic qPCR

Validation by an independent assay, i.e. quantitative polymerase chain reaction (qPCR), was obtained for de novo and/or unreported CNVs identified in PS patients (Additional file 1) according to established protocols [16]. Briefly, DNA from the patients was analyzed together with DNA from one healthy adult used as control. Primers were designed to amplify a region lying inside the deletion or duplication and one region flanking the CNV. A region on chromosome 12 encompassing the *GAPDH* gene (NM_002046.3) was used as internal control to determine copy number and normalize primer efficiency (primer sequences are available on request). qPCR was performed using the iCycler (Biorad, Hercules, CA) with Sybr Green, and the comparative DDCt method as previously described [16, 17].

### Bioinformatics and network analysis of genes included within CNVs

We searched for mutations affecting the identified CNV-genes, and possibly associated to genetic disorders and/or anomalous phenotype, through available public databases: OMIM, PubMed, UCSC, GeneCard (http://www.genecards.org/), ClinVar (https://www.ncbi.nlm.nih.gov/clinvar/).

We used GeneCodis3 [18] to unveil enrichment of annotations. Genomic coordinates of altered regions were used to retrieve CNV overlapping genes from the hg19 RefSeq track of the UCSC genome browser. All the genes obtained from either duplicated or deleted regions were used as input in GeneCodis (Additional file 2). This tool allows the classification of genes according to their putative biological function by screening the Gene Ontology (GO), OMIM, Panther Pathway, and KEGG Pathway. In the analysis, the hypergeometric test was applied followed by the false discovery rate correction (FDR) with a cut-off of 5% to determine which annotations were significantly enriched. For GO analysis, various hierarchical levels of the annotation data structure were used. A graphical representation of the GeneCodis analysis results and of the possible interrelationship among the CNV-genes was obtained by using Cytoscape tool [19].

## Results

### Clinical phenotypes

Clinical data from 120 sporadic PS patients, 115 new and 5 already reported [1, 8, 9] were collected and their clinical features summarised in Table 1. Rarely, PS patients can show associated anomalies involving other structures/organs [20]. Overall, no dysmorphic signs or other anomalies were observed in these patients but one, who presented with associated Möbius syndrome features. This patient was referred to the Medical Genetics Unit of Istituto Gaslini Genoa at the age of 9 months showing the following anomalies: weight and head circumference < 3^rd^ centile; length at 5^th^ centile; right facial nerve paralysis; epicanthic folds; micrognatia and cleft palate (Pierre Robin sequence, MIM261800); right abducens nerve paralysis; right auditory nerve paralysis; right upper limb hypoplasia; flexion deformity of the left elbow and the left wrist.

### Genomic rearrangements identified in Poland syndrome patients

Karyotyping and array-CGH analyses were performed in all patients. This survey led to the identification of 14 different heterozygous chromosome anomalies in 14 different patients, notably the same alteration was never reported in more than one patient (Table 2). The duplication of patient PS14 and the deletion of patient PS15 overlapped the same 1.8Mbp region on chromosome 16p13.1 causing a dosage imbalance of the same 12 RefSeq genes. Smaller deletions affecting chromosome 16p13.1 were found in two in-house controls, and chromosome 16p13.1 deletions and 16p13.1 duplications were reported in patients with intellectual disabilities [21] and autism [22], respectively, without any PS-associated clinical sign. Except for CNVs involving chromosome 16p13.1 region, CNV data of PS patients and in-house controls did not reveal any shared CNVs. The deletion of patient PS6 overlaps the *AUTS2* gene, a susceptibility candidate gene for autism that is not related to PS-involved tissues.

**Table 2:** Genomic deletions and duplications detected in 19 patients with Poland syndrome

Patients ID	Gender	Chr. band	Coordinates Hg19	Size (Mbp)	CNV	Possible candidate genes^a^	Inh	Pectoralis muscle phenotype (L/R)/Additional anomalies (L/R)	Previous reports
2	F	5p15.2	chr5:13266637-14011580	0.74	Dup	*DNAH5, TRIO*	Mat	hypoplasia (L)/upper limb hypoplasia (L)	-
3	M	5p14.3	chr5:22187485-22740287	0.55	Dup	*CDH12*	de novo	hypoplasia (R)/hand hypoplasia (R)	-
4^b^	F	5p14.1-p13.3	chr5:27656463-29650802	1.99	Del	*CDH6, CDH9*	Mat	hypoplasia (R)	-
5	M	6q21-q22.1	chr6:111777947-116488007	4.71	Del	*REV3L, FYN, WISP3, LAMA4, MARCKS, FRK, COL10A1*	Nd	hypoplasia (R)/scoliosis/pectus excavatum/intellectual disability	(22)
6	F	7q11.22	chr7:70182803-70223737	0.04	Del	*-*	de novo	agenesis (L)/brachysyndactyly (L)/ASD, pectus excavatum	-
7	F	9p24.2-p24.1	chr9:4152060-4627624	0.48	Dup	*GLIS3*	Mat	agenesis (L)/upper limb hypoplasia (L)	-
9	F	11q12.3	chr11:63185662-63342369	0.16	Del	*HRASLS5, RARRES3, HRASLS2, PLA2G16*	de novo	hypoplasia (R)/hand hypoplaisa, vertebral defects	(8)
10	M	11p14.1	chr11:28131098-28349712	0.22	Del	*KIF18A*	Mat	hypoplasia (R)	-
11	F	12q21.31-q21.32	chr12:86018191-87432656	1.41	Dup	*NTS*	Pat	hypoplasia (R)/brachydactyly	-
12	M	13q12.11-q12.12	chr13:22840054-24890143	2.05	Del	*SGCG, SACS, SPATA13*	Mat	agenesis (R)/brachysyndactyly (R), pectus excavatum	-
14	F	16p13.11-p12.3	chr16:15256686-18546759	3.29	Dup	*NDE1, MYH11, ABCC6, XYLT1*	Not mat	agenesis (R)/upper limb hypoplasia (R)	-
15	M	16p13.11-p12.3	chr16:15131723-16305736	1.17	Del	*RRN3, NDE1, MYH11, ABCC6*	Nd	hypoplasia (R), pectus carinatum	-
16	F	16q22.3-q23.1	chr16:74087653-74519724	0.43	Dup	*PSMD7, GLG1*	Mat	agenesis (L)/upper limb hypoplasia (L), rib defects	(1)
20	M	Xp11.22	chrX:53666883-54056673	0.39	Dup	*HUWE1*	Mat	hypoplasia (R)	(1)

^a^Genes previously implicated in: muscle/limb/skeletal structure and/or development; blood vessel structure and/or development; cell junction organization; cell division. ^b^In this case the deletion does not encompass any genes, however, since it overlaps 5′ regions of two genes, *CDH6, CDH9*, these genes were both enclosed in this table as their expression could be impaired by the deletion. Inh = inheritance

The parental origin was assessed for 11 of identified CNVs: 3 occurred *de novo*, eight were inherited from a healthy parent (seven maternal, one paternal). Clinical evaluation excluded the presence of any thoracic anomalies in the parents of all PS patients, indeed, the parents of patients PS11 and PS12, suspected to present a slight form of pectoralis muscle hypoplasia, were further examined by ultrasound analysis that confirmed a normal phenotype for all of them.

One patient (PS2) carried a duplication, dup(5)(p15.2), resulting from translocation 46, XX, t(5;11)(p15.1;q21) inherited from his unaffected mother. This was the only chromosome anomaly identified by standard karyotyping, one additional PS patient presented with 47,XXY karyotype associated with Klinefleter syndrome. Of the fourteen identified anomalies, except for 16p13.11-p12.3 deletion and duplication in patient PS14 and PS15 respectively, 12 were not present in DGV or differed from the reported CNVs by at least 100 kbp and/or involved at least one more gene. No newly identified CNVs matched those reported in DECIPHER or ClinGen.

### Annotation of genes within CNVs

Based on the RefSeq database, 119 genes were identified in either duplicated or deleted regions (CNV-genes), including CNV-encompassed genes, genes interrupted by CNVs, and the two genes flanking CNVs on both sides. These last genes were included in the study according to the hypothesis that CNVs could impair their expression by removing or duplicating or separating expression regulatory elements from the coding sequences (Additional file 2). We investigated each CNV-gene for mutations associated to known disorder and/or anomalous phenotype through the use of available public genetic databases. Only one gene, *REV3L*, resulted to be recently described as associated to one patient with PS features [23]. Thirteen genes were known disease genes reported in OMIM.

To gain further insight into the deleterious effects of mutations involving all CNV-genes, we performed a complete analysis of phenotypes resulting from inactivation of CNV-gene murine and zebrafish orthologs. For 34 genes, at least one murine mutant has been reported and phenotypically characterized. Most reported mouse mutants presented defects in tissues different from those affected in PS. For each patient, CNV-genes that could be considered as PS candidate genes on the basis of their expression pattern or molecular function are showed in Table 2.

### Bioinformatic analysis of genes included within CNVs

**Fig. 1:**
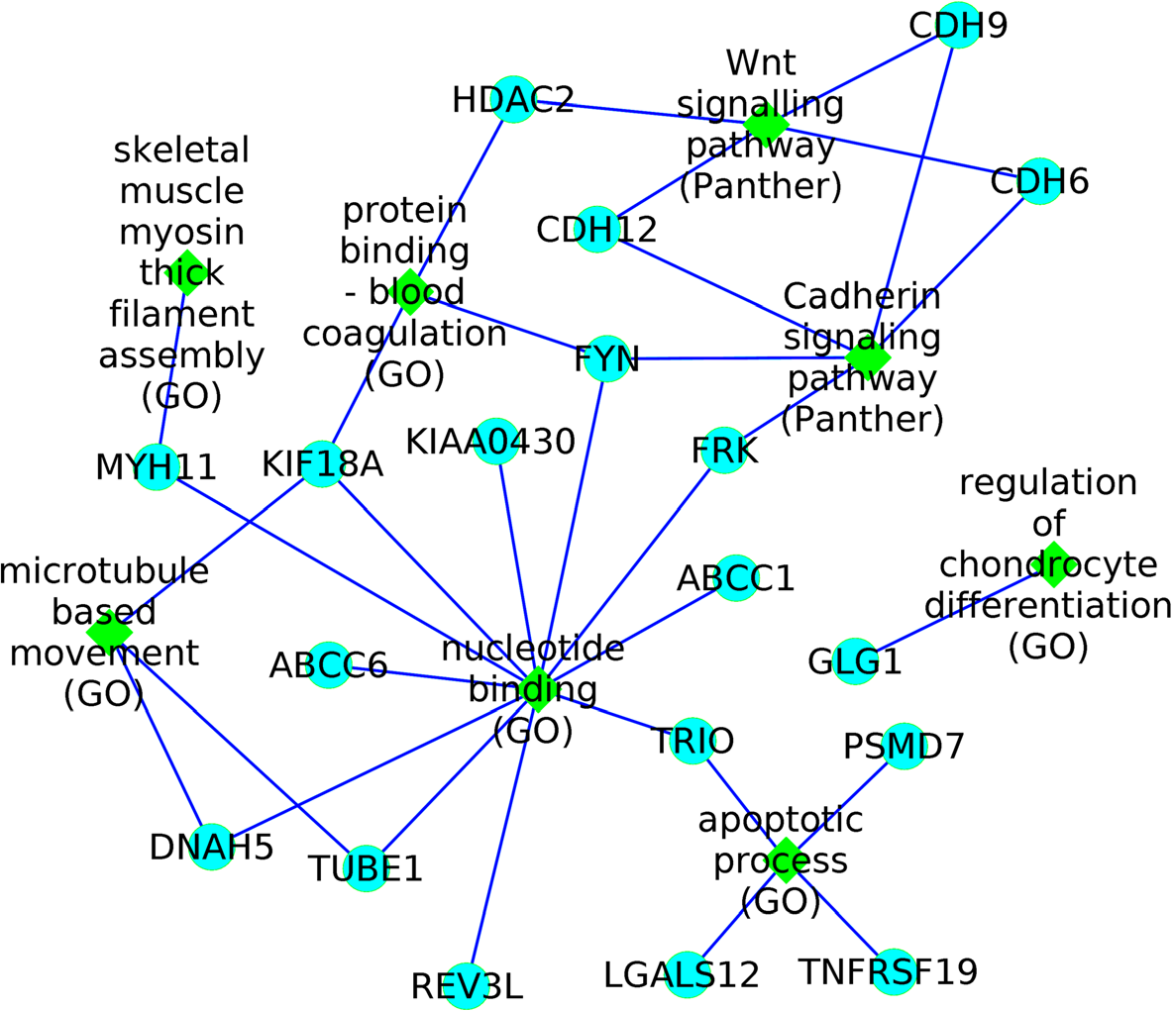
Graphical representation of the possible interrelationship among the CNV-genes. In this figure, CNV-genes (*light blue rounded nodes*) and enriched categories (*green diamonds*) identified by GeneCodis analysis and their possible relantionships are visualized by Cytoscape tool

All CNV-genes were analyzed by GeneCodis to search for a significant enrichment of annotations, which can be used as functional descriptor of the biological processes involved in PS. Sixty-one of the total genes were annotated using GeneCodis, unmapped genes consisted of poorly annotated non-coding RNAs. We identified significant enrichment of 8 biological categories (Table 3, Fig. 1), some of them, as those concerning mechanisms of chondrocyte and/or skeletal muscle development, blood coagulation, and DNA binding and apoptotic processes, relevant as candidate mechanisms contributing to PS development.

**Table 3:** Biological processes associated with genes rearranged in patients with PS

Genes	Annotations	List size^a^	Reference Support^b^	Reference size^c^	Hyp_c^d^
FYN, CDH6, CDH9, FRK, CDH12	Panther P00012: Cadherin signaling pathway (modular enrichment)	61	140	34208	7.40x10^-5^
FYN, TRIO, KIF18A, MYH11, FRK, TUBE1, ABCC6, KIAA0430, REV3L, DNAH5, ABCC1	GO 0000166: nucleotide binding (MF) (modular enrichment)	61	2120	34208	3.70x10^-3^
CDH6,HDAC2, CDH9,CDH12	Panther P00057: Wnt signaling pathway (modular enrichment)	61	280	34208	4.05x10^-3^
FYN, KIF18A, HDAC2	GO 0005515: protein binding (MF); GO 0007596: blood coagulation (BP) (modular enrichment)	61	251	34208	1.92x10^-2^
TRIO, LGALS12, PSMD7, TNFRSF19	GO:0006915, apoptotic process (BP) (modular enrichment)	61	594	34208	3.02x10^-2^
KIF18A, TUBE1, DNAH5	GO 0007018, microtubule-based movement (BP)	61	90	34208	2.88x10^-2^
GLG1	GO 0032330: regulation of chondrocyte differentiation (BP)	61	3	34208	4.17x10^-2^
MYH11	GO 0030241, skeletal muscle myosin thick filament assembly (BP)	61	3	34208	4.17x10^-2^

*Abbreviations*: ^a^Total number of genes in the input CNV list. ^b^Number of annotated genes in the reference list. ^c^Total number of genes in the reference list. ^d^Hyp_c = Corrected hypergeometric pValue using FDR procedure with a cut-off of 5% GeneCodis. BP = biological processes. MF = molecular function

## Discussion

Here we present the first comprehensive survey of CNVs in PS and provide a catalog of CNVs and candidate genes possibly implicated in the PS phenotype. Most of the novel rearrangements identified in this study were transmitted from unaffected parents. This is expected on the basis of incomplete penetrance observed in familial cases [1] and indicates that these CNVs may act as susceptibility alleles rather than direct causes of PS. In our cohort of patients, CNVs involved different genomic regions and different genes, which indicates that each identified CNV can account for single specific cases. This lack of overlap can be due to a sampling issue, or one may hypothesize that, if more than one CNV is related to the disease, the genes involved in these CNVs are members of the same family and/or belong to the same pathways. To unveil possible interplays among identified CNV-genes, we performed a comprehensive bioinformatic analysis of CNVs identified in our patients (Tab. 2). This analysis showed significant enrichment of proteins involved in cell adhesion, blood coagulation, chondrogenesis, asymmetric development, skeletal muscle structure, and nucleotide binding (Table 3). For each category and/or according to their expression pattern and function, we present the most relevant genes based on their hypothetical role in PS. The complete lack of *LAMA4* caused in mice hemorrhages associated with capillary defects, adipose tissue alteration, and motor control impairment [24, 25]. These findings suggest that the deletion of *LAMA4*, by inducing hemorrhages during embryonic development, could cause a variety of defects, possibly including those observed in PS patients. This hypothesis is in line with that of disruption of blood supply during development as causative of PS [5, 6]. The *MYH11* gene, encoding the contractile protein myosin, was found duplicated in patient PS14 and deleted in patient PS15, suggesting it could be a dosage-sensitive gene. *MYH11* missense mutations cause a dominant form of thoracic aortic aneurysm and/or aortic dissection (AAT4, MIM132900) and *Myh11* null mice exhibit dilated cardiomyopathy [26]. No pectoralis muscle anomalies nor PS-associated bone defects have been reported in AAT4 patients or described in *Myh11* null mice. In our study, the cadherin signaling pathway resulted as the most significant among all analyzed categories and, notably, the *CDH12* spanned the breakpoint of a duplication originating de novo in one patient. Neither hereditary disease nor animal models are known for *CDH12. Glg1* null mice were found to die shortly after birth and about 30% of the few surviving mice had bone defects [27]. Patients with deletion of *DNAH5* exhibit randomization of left-right body asymmetry as situs inversus, or partial transposition of the viscera as isolated dextrocardia. Asymmetry is also a major feature of PS and the combination of PS and isolated dextrocardia was described in 11% of PS patients [28]. *DNAH5* was duplicated in patient PS2 who presented asymmetric development of pectoralis muscles and upper limbs, although without dextrocardia. No data have been reported on the effects of enhanced *DNAH5* expression, for instance due to gene duplication, so we cannot exclude that both mechanisms, i.e. deficiency as well as enhancement of *DNAH5* function, may induce asymmetric development. Asymmetric development was also observed in mice carrying inactivation of the *Rrn3* gene [29]. The *SGCG* gene is associated with a recessive form of limb-girdle muscular dystrophy (LGMD2C, MIM253700). Of note, both PS and LGMD2C affect muscles of the limb-girdle.

An attractive candidate gene is *TRIO,* whose complete absence in mice causes embryonic lethality associated with abnormal development of skeletal muscle and neural tissues. Indeed, *Trio* deficiency caused a specific defect of myogenesis resulting in anomalies of skeletal muscle formation [30].

In fact, after consulting available genetic databases, OMIM, and PubMed, no mutations in any of the above mentioned genes have been previously reported as causing a phenotype resembling that showed by PS patients, except for one, the *REV3L* gene [23].

Mutations in the *REV3L* gene have been recently associated with Möbius syndrome (MBS) [23], a rare congenital cranial dysinnervation disorder characterized by facial palsy and variable other congenital anomalies, including pectoralis muscle hypoplasia. Thomas-Roca and collaborators showed that mutations in *REV3L* are responsible for a proportion of MBS patients with highly variable clinical features and no obvious genotype–phenotype correlations. Accordingly, the pectoralis muscle agenesis was observed in only one among the three MBS patients carrying heterozygous *REV3L* mutations [23]. The *REV3L* gene encodes a protein acting as catalytic subunit of DNA polymerase z, an error-prone DNA polymerase with a key role in replication of damaged DNA [31, 32]. In the absence of *REV3L* [33], unrepaired DNA damage triggers apoptosis via the accumulation of double-stranded DNA breaks. To explain the variability of phenotypes associated to the identified *REV3L* mutations in the MBS, Thomas-Roca and collaborators hypothesized that *REV3L* deficiency may result in a stochastic ablation of cell lineages during embryonic development, caused by replicative stress at endogenous DNA lesions and consequent DNA damage responses. According to this hypothesis, the appearance of the described PS-MBS associated phenotype in one *REV3L* mutation carrier may represent a very rare stochastic event. The description of a case carrying a deletion affecting the *REV3L* gene and showing a complex phenotype including PS without any apparent MBS signs (patient PS5) may support the hypothesis of mechanisms leading *REV3L* mutations to cause variable phenotypes but also seems to support a direct role of *REV3L* gene in pectoralis muscle development.

## Conclusion

Data obtained by standard karyotype and array-CGH analyses in our cohort of 120 patients suggest that chromosome anomalies, duplications and deletions, are a rare cause of PS. Most of identified CNVs are inherited by unaffected parents, thus suggesting they could act as modifiers and represent risk factors of PS. Genes overlapped by the identified CNVs are enriched in functional categories and pathways involved in cell-cell adhesion, DNA binding and apoptosis processes, suggesting these processes as playing a role in PS development and indicating the identified genes as candidates for further evaluation in functional studies or mutation screening in other patients by direct sequencing or exome sequencing.

## Additional files

Additional file 1:
**Figure S1.** Quantitative polymerase chain reaction. In the Figure, examples of qPCR results obtained from three PS patients carrying three different CNVs are shown. A) Patient PS16: primers were designed to amplify a region encompassed by the duplication (chr16:74,313,856-74,313,966) and a 5′ flanking region (chr16:73,857,457-73,857,556); B) patient PS3: primers were designed to amplify a region encompassed by the duplication (chr5:22,539,039-22,539,170) and a 5′ flanking region (chr5:21,756,984-21,757,122); C) Patient PS10: primers were designed to amplify a region encompassed by the deletion (chr11:28,126,119-28,126,268) and a 5′ flanking region (chr11:28,075,866-28,076,003). Fold change of about 1 is expected for a diploid sample, about 0.5 for a haploid sample, and about 2.0 for a triploid sample. ctr: genomic DNA from one healthy adult used as control. (TIF 1168 kb)
Additional file 2:
**Table S2.** Annotated CNV-genes of PS patients. DUP = gene encompassed by a duplication; DEL = gene encompassed by a deletion; CNV-flanking gene = gene flanking a CNV; DUP-INTERRUPT = gene overlapping a duplication breakpoint. (DOCX 17.5 kb)

## Abbreviations

Array-CGH: Array-comparative genomic hybridization
CNVs: Copy number variations
PS: Poland Syndrome
qPCR: Quantitative polymerase chain reaction

## References

[1] Baban A, Torre M, Costanzo S, Gimelli S, Bianca S, Divizia MT, Senes FM, Garavelli L, Rivieri F, Lerone M (2012). Familial Poland anomaly revisited. Am J Med Genet A.

[2] Catena N, Divizia MT, Calevo MG, Baban A, Torre M, Ravazzolo R, Lerone M, Senes FM (2012). Hand and upper limb anomalies in Poland syndrome: a new proposal of classification. J Pediatr Orthop.

[3] Freire-Maia N, Chautard EA, Opitz JM, Freire-Maia A, Quelce-Salgado A (1973). The Poland syndrome-clinical and genealogical data, dermatoglyphic analysis, and incidence. Hum Hered.

[4] McGillivray BC, Lowry RB (1977). Poland syndrome in British Columbia: incidence and reproductive experience of affected persons. Am J Med Genet.

[5] Bavinck JN, Weaver DD (1986). Subclavian artery supply disruption sequence: hypothesis of a vascular etiology for Poland, Klippel-Feil, and Mobius anomalies. Am J Med Genet.

[6] Beer GM, Kompatscher P, Hergan K (1996). Poland’s syndrome and vascular malformations. Br J Plast Surg.

[7] Valasek P, Theis S, DeLaurier A, Hinits Y, Luke GN, Otto AM, Minchin J, He LW, Christ B, Brooks G (2011). Cellular and molecular investigations into the development of the pectoral girdle. Dev Biol.

[8] Vaccari CM, Romanini MV, Musante I, Tassano E, Gimelli S, Divizia MT, Torre M, Morovic CG, Lerone M, Ravazzolo R (2014). De novo deletion of chromosome 11q12.3 in monozygotic twins affected by Poland Syndrome. BMC Med Genet.

[9] Tassano E, Mirabelli-Badenier M, Veneselli E, Puliti A, Lerone M, Vaccari CM, Morana G, Porta S, Gimelli G, Cuoco C (2015). Clinical and molecular characterization of a patient with interstitial 6q21q22.1 deletion. Mol Cytogenet.

[10] Georgieva L, Rees E, Moran JL, Chambert KD, Milanova V, Craddock N, Purcell S, Sklar P, McCarroll S, Holmans P (2014). De novo CNVs in bipolar affective disorder and schizophrenia. Hum Mol Genet.

[11] Silversides CK, Lionel AC, Costain G, Merico D, Migita O, Liu B, Yuen T, Rickaby J, Thiruvahindrapuram B, Marshall CR (2012). Rare copy number variations in adults with tetralogy of Fallot implicate novel risk gene pathways. PLoS Genet.

[12] Striano P, Coppola A, Paravidino R, Malacarne M, Gimelli S, Robbiano A, Traverso M, Pezzella M, Belcastro V, Bianchi A (2012). Clinical significance of rare copy number variations in epilepsy: a case-control survey using microarray-based comparative genomic hybridization. Arch Neurol.

[13] Tassano E, Gimelli S, Divizia MT, Lerone M, Vaccari C, Puliti A, Gimelli G (2015). Thrombocytopenia-absent radius (TAR) syndrome due to compound inheritance for a 1q21.1 microdeletion and a low-frequency noncoding RBM8A SNP: a new familial case. Mol Cytogenet.

[14] Verbitsky M, Sanna-Cherchi S, Fasel DA, Levy B, Kiryluk K, Wuttke M, Abraham AG, Kaskel F, Kottgen A, Warady BA (2015). Genomic imbalances in pediatric patients with chronic kidney disease. J Clin Invest.

[15] Miller DT, Adam MP, Aradhya S, Biesecker LG, Brothman AR, Carter NP, Church DM, Crolla JA, Eichler EE, Epstein CJ (2010). Consensus statement: chromosomal microarray is a first-tier clinical diagnostic test for individuals with developmental disabilities or congenital anomalies. Am J Hum Genet.

[16] Rossi PIA, Vaccari CM, Terracciano A, Doria-Lamba L, Facchinetti S, Priolo M, Ayuso C, De Jorge L, Gimelli S, Santorelli FM (2010). The metabotropic glutamate receptor 1, GRM1: Evaluation as a candidate gene for inherited forms of cerebellar ataxia. J Neurol.

[17] Livak KJ, Schmittgen TD (2001). Analysis of relative gene expression data using real-time quantitative PCR and the 2(T)(-Delta Delta C) method. Methods.

[18] Tabas-Madrid D, Nogales-Cadenas R, Pascual-Montano A (2012). GeneCodis3: a non-redundant and modular enrichment analysis tool for functional genomics. Nucleic Acids Res.

[19] Smoot ME, Ono K, Ruscheinski J, Wang PL, Ideker T (2011). Cytoscape 2.8: new features for data integration and network visualization. Bioinformatics.

[20] Silengo M, Lerone M, Seri M, Boffi P (1999). Lower extremity counterpart of the Poland syndrome. Clin Genet.

[21] de Kovel CG, Trucks H, Helbig I, Mefford HC, Baker C, Leu C, Kluck C, Muhle H, von Spiczak S, Ostertag P (2010). Recurrent microdeletions at 15q11.2 and 16p13.11 predispose to idiopathic generalized epilepsies. Brain.

[22] Stefansson H, Meyer-Lindenberg A, Steinberg S, Magnusdottir B, Morgen K, Arnarsdottir S, Bjornsdottir G, Walters GB, Jonsdottir GA, Doyle OM (2014). CNVs conferring risk of autism or schizophrenia affect cognition in controls. Nature.

[23] Tomas-Roca L, Tsaalbi-Shtylik A, Jansen JG, Singh MK, Epstein JA, Altunoglu U, Verzijl H, Soria L, van Beusekom E, Roscioli T (2015). De novo mutations in PLXND1 and REV3L cause Mobius syndrome. Nat Commun.

[24] Thyboll J, Kortesmaa J, Cao R, Soininen R, Wang L, Iivanainen A, Sorokin L, Risling M, Cao Y, Tryggvason K (2002). Deletion of the laminin alpha4 chain leads to impaired microvessel maturation. Mol Cell Biol.

[25] Vaicik MK, Thyboll Kortesmaa J, Moverare-Skrtic S, Kortesmaa J, Soininen R, Bergstrom G, Ohlsson C, Chong LY, Rozell B, Emont M (2014). Laminin alpha4 deficient mice exhibit decreased capacity for adipose tissue expansion and weight gain. PLoS One.

[26] Morano I, Chai GX, Baltas LG, Lamounier-Zepter V, Lutsch G, Kott M, Haase H, Bader M (2000). Smooth-muscle contraction without smooth-muscle myosin. Nat Cell Biol.

[27] Miyaoka Y, Tanaka M, Imamura T, Takada S, Miyajima A (2010). A novel regulatory mechanism for Fgf18 signaling involving cysteine-rich FGF receptor (Cfr) and delta-like protein (Dlk). Development.

[28] Torre M, Baban A, Buluggiu A, Costanzo S, Bricco L, Lerone M, Bianca S, Gatti GL, Senes FM, Valle M (2010). Dextrocardia in patients with Poland syndrome: phenotypic characterization provides insight into the pathogenesis. J Thorac Cardiovasc Surg.

[29] Yuan X, Zhou Y, Casanova E, Chai M, Kiss E, Grone HJ, Schutz G, Grummt I (2005). Genetic inactivation of the transcription factor TIF-IA leads to nucleolar disruption, cell cycle arrest, and p53-mediated apoptosis. Mol Cell.

[30] O’Brien SP, Seipel K, Medley QG, Bronson R, Segal R, Streuli M (2000). Skeletal muscle deformity and neuronal disorder in Trio exchange factor-deficient mouse embryos. Proc Natl Acad Sci U S A.

[31] Lin W, Wu X, Wang Z (1999). A full-length cDNA of hREV3 is predicted to encode DNA polymerase zeta for damage-induced mutagenesis in humans. Mutat Res.

[32] Van Sloun PP, Varlet I, Sonneveld E, Boei JJ, Romeijn RJ, Eeken JC, De Wind N (2002). Involvement of mouse Rev3 in tolerance of endogenous and exogenous DNA damage. Mol Cell Biol.

[33] Schenten D, Kracker S, Esposito G, Franco S, Klein U, Murphy M, Alt FW, Rajewsky K (2009). Pol zeta ablation in B cells impairs the germinal center reaction, class switch recombination, DNA break repair, and genome stability. J Exp Med.

