# Supplementary figures and images for: Assessment of copy number variations in 120 patients with Poland syndrome
Source: BMC Med Genet. 2016 Nov 25;17:89. doi: 10.1186/s12881-016-0351-x (PMC5123256; doi:10.1186/s12881-016-0351-x)

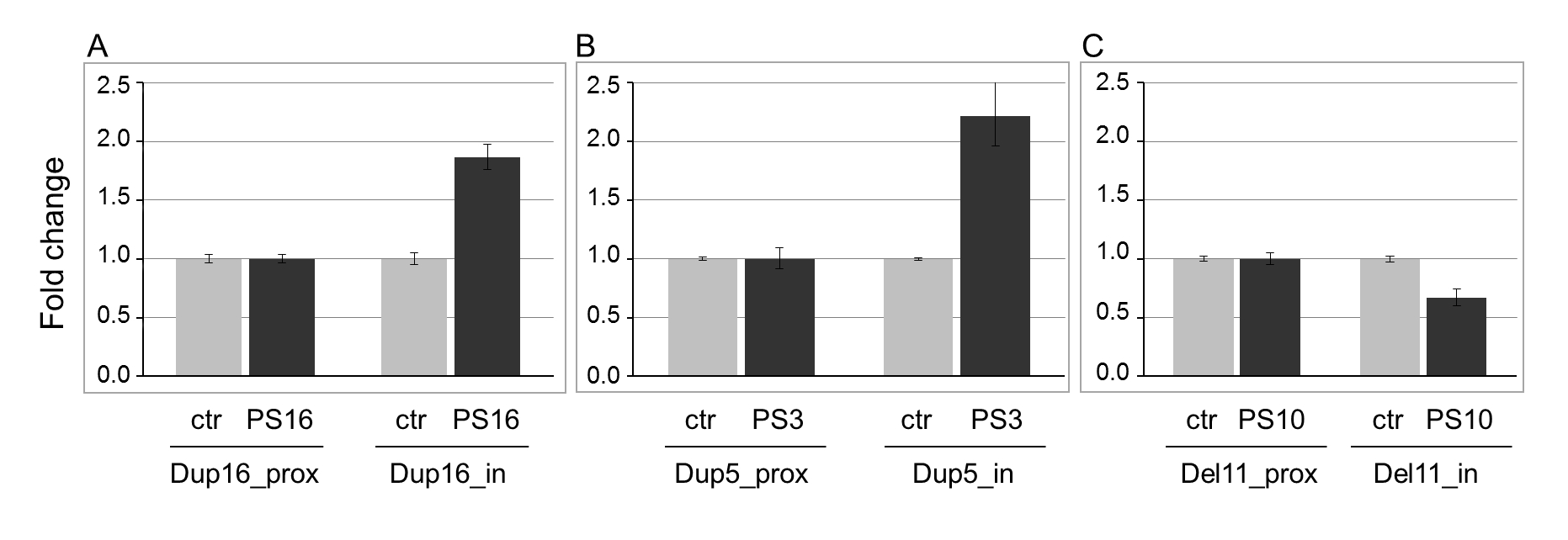

Supplement: Additional file 1: — Figure S1. Quantitative polymerase chain reaction. In the Figure, examples of qPCR results obtained from three PS patients carrying three different CNVs are shown. A) Patient PS16: primers were designed to amplify a region encompassed by the duplication (chr16:74,313,856-74,313,966) and a 5′ flanking region (chr16:73,857,457-73,857,556); B) patient PS3: primers were designed to amplify a region encompassed by the duplication (chr5:22,539,039-22,539,170) and a 5′ flanking region (chr5:21,756,984-21,757,122); C) Patient PS10: primers were designed to amplify a region encompassed by the deletion (chr11:28,126,119-28,126,268) and a 5′ flanking region (chr11:28,075,866-28,076,003). Fold change of about 1 is expected for a diploid sample, about 0.5 for a haploid sample, and about 2.0 for a triploid sample. ctr: genomic DNA from one healthy adult used as control. (TIF 1168 kb) [file 12881_2016_351_MOESM1_ESM.tif]
