# Supplementary material for: Assessment of copy number variations in 120 patients with Poland syndrome
Source: BMC Med Genet. 2016 Nov 25;17:89. doi: 10.1186/s12881-016-0351-x (PMC5123256; doi:10.1186/s12881-016-0351-x)
Supplement: Additional file 2: — Table S2. Annotated CNV-genes of PS patients. DUP = gene encompassed by a duplication; DEL = gene encompassed by a deletion; CNV-flanking gene = gene flanking a CNV; DUP-INTERRUPT = gene overlapping a duplication breakpoint. (DOCX 17.5 kb) [file 12881_2016_351_MOESM2_ESM.docx]

**Table S1. Annotated CNV-genes of PS patients**

| **#name (RefSeq)** | **chrom** | **Symbol** | **Status** |
| --- | --- | --- | --- |
| NM_001369 | chr5 | DNAH5 | DUP |
| NR_033383 | chr5 | LINC01194 | DUP-INTERRUPT |
| NM_007118 | chr5 | TRIO | DUP-INTERRUPT |
| NM_001317228 | chr5 | CDH12 | DUP-INTERRUPT |
| NR_003921 | chr5 | PMCHL1 | DUP/CNV-flanking gene |
| NM_016279 | chr5 | CDH9 | CNV-flanking gene |
| NR_038848 | chr5 | LINC01021 | CNV-flanking gene |
| NR_134265 | chr5 | LOC105374698 | DEL |
| NR_033961 | chr5 | LSP1P3 | DEL |
| NR_109948 | chr5 | LOC101929645 | DEL |
| NR_130777 | chr5 | LOC101929660 | DEL |
| NR_104628 | chr5 | LOC101929681 | DEL |
| NR_134264 | chr5 | LOC105374704 | CNV-flanking gene |
| NM_004932 | chr5 | CDH6 | CNV-flanking gene |
| NM_001286431 | chr6 | REV3L | DEL |
| NR_034110 | chr6 | TRAF3IP2-AS1 | DEL |
| NM_001164283 | chr6 | TRAF3IP2 | DEL |
| NM_153048 | chr6 | FYN | DEL |
| NR_125354 | chr6 | WISP3 | DEL |
| NM_016262 | chr6 | TUBE1 | DEL |
| NM_001033564 | chr6 | FAM229B | DEL |
| NM_001105206 | chr6 | LAMA4 | DEL |
| NR_121193 | chr6 | LOC101927640 | DEL |
| NM_001013734 | chr6 | RFPL4B | DEL |
| NR_125844 | chr6 | LOC101927686 | DEL |
| NM_002356 | chr6 | MARCKS | DEL |
| NR_038863 | chr6 | LINC01268 | DEL |
| NR_027060 | chr6 | FLJ34503 | DEL |
| NR_073443 | chr6 | HDAC2 | DEL |
| NR_125845 | chr6 | LOC101927768 | DEL |
| NM_153612 | chr6 | HS3ST5 | DEL |
| NR_134602 | chr6 | LOC105377962 | DEL |
| NM_002031 | chr6 | FRK | DEL |
| NR_027338 | chr6 | TPI1P3 | DEL |
| NM_152729 | chr6 | NT5DC1 | DEL |
| NM_000493 | chr6 | COL10A1 | DEL |
| NM_001042413 | chr9 | GLIS3 | DUP-INTERRUPT |
| NM_004170 | chr9 | SLC1A1 | DUP |
| NM_001039395 | chr9 | SPATA6L | DUP-INTERRUPT |
| NM_080866 | chr11 | SLC22A9 | CNV-flanking gene |

| **#name (RefSeq)** | **chrom** | **Symbol** | **Status** |
| --- | --- | --- | --- |
| NM_001146728 | chr11 | HRASLS5 | DEL |
| NM_033101 | chr11 | LGALS12 | DEL |
| NM_004585 | chr11 | RARRES3 | DEL |
| NM_017878 | chr11 | HRASLS2 | DEL |
| NM_007069 | chr11 | PLA2G16 | DEL |
| NM_031217 | chr11 | KIF18A | CNV-flanking gene |
| NM_001113528 | chr11 | METTL15 | DEL |
| NM_005447 | chr12 | RASSF9 | DUP |
| NM_006183 | chr12 | NTS | DUP |
| NM_013244 | chr12 | MGAT4C | DUP |
| NR_103810 | chr13 | LINC00540 | DEL |
| NR_033774 | chr13 | BASP1P1 | DEL |
| NM_000231 | chr13 | SGCG | DEL |
| NM_014363 | chr13 | SACS | DEL |
| NR_103450 | chr13 | SACS-AS1 | DEL |
| NR_038995 | chr13 | LINC00327 | DEL |
| NM_001204459 | chr13 | TNFRSF19 | DEL |
| NM_005932 | chr13 | MIPEP | DEL |
| NM_001014442 | chr13 | C1QTNF9B-AS1 | DEL |
| NR_104426 | chr13 | C1QTNF9B | DEL |
| NR_073430 | chr13 | ANKRD20A19P | DEL |
| NR_104595 | chr13 | SPATA13 | DEL |
| NR_031753 | chr13 | MIR2276 | DEL |
| NR_046531 | chr13 | SPATA13-AS1 | DEL |
| NM_001303138 | chr13 | C1QTNF9 | DEL |
| NM_001277325 | chr16 | NPIPA5 | DUP |
| NM_001128423 | chr16 | MPV17L | DUP |
| NM_033201 | chr16 | C16orf45 | DUP |
| NM_014647 | chr16 | KIAA0430 | DUP |
| NR_106761 | chr16 | MIR6506 | DUP |
| NM_001143979 | chr16 | NDE1 | DUP |
| NR_030159 | chr16 | MIR484 | DUP |
| NM_022844 | chr16 | MYH11 | DUP |
| NR_130755 | chr16 | FOPNL | DUP |
| NM_004996 | chr16 | ABCC1 | DUP |
| NM_001171 | chr16 | ABCC6 | DUP |
| NM_001004067 | chr16 | NOMO3 | DUP |
| NR_128709 | chr16 | MIR3179-4 | DUP |
| NR_036145 | chr16 | MIR3179-3 | DUP |
| NR_036143 | chr16 | MIR3179-2 | DUP |
| NR_036140 | chr16 | MIR3179-1 | DUP |

| **#name (RefSeq)** | **chrom** | **Symbol** | **Status** |
| --- | --- | --- | --- |
| NR_037442 | chr16 | MIR3670-1 | DUP |
| NR_049832 | chr16 | MIR3670-2 | DUP |
| NR_128712 | chr16 | MIR3670-3 | DUP |
| NR_128713 | chr16 | MIR3670-4 | DUP |
| NR_036141 | chr16 | MIR3180-1 | DUP |
| NR_036144 | chr16 | MIR3180-3 | DUP |
| NR_036142 | chr16 | MIR3180-2 | DUP |
| NR_036447 | chr16 | PKD1P1 | DUP |
| NR_103772 | chr16 | LOC100288162 | DUP |
| NR_106970 | chr16 | MIR6511A3 | DUP |
| NR_106766 | chr16 | MIR6511A1 | DUP |
| NR_106969 | chr16 | MIR6511A2 | DUP |
| NR_106971 | chr16 | MIR6511A4 | DUP |
| NR_107061 | chr16 | MIR6770-3 | DUP |
| NR_107060 | chr16 | MIR6770-2 | DUP |
| NR_106828 | chr16 | MIR6770-1 | DUP |
| NM_001282511 | chr16 | NPIPA8 | DUP |
| NM_001282507 | chr16 | NPIPA7 | DUP |
| NM_022166 | chr16 | XYLT1 | DUP |
| NR_135179 | chr16 | LOC102723692 | DUP |
| NM_001004060 | chr16 | NOMO2 | DUP |
| NM_001285447 | chr16 | PDXDC1 | DEL |
| NM_173474 | chr16 | NTAN1 | DEL |
| NM_001301064 | chr16 | RRN3 | DEL |
| NR_125434 | chr16 | LOC100505915 | DEL |
| NR_123721 | chr16 | PKD1P6-NPIPP1 | DEL |
| NR_106775 | chr16 | MIR6511B1 | DEL |
| NR_106965 | chr16 | MIR6511B2 | DEL |
| NR_037466 | chr16 | MIR3180-4 | DEL |
| NR_104657 | chr16 | LOC101928035 | DUP |
| NM_002811 | chr16 | PSMD7 | DUP |
| NR_026950 | chr16 | LOC283922 | DUP |
| NM_001306094 | chr16 | NPIPB15 | DUP |
| NR_135170 | chr16 | LOC105376772 | DUP |
| NM_001011880 | chr16 | CLEC18B | DUP |
| NR_027264 | chr16 | GLG1 | DUP-INTERRUPT |
| NM_031407 | chrX | HUWE1 | DUP-INTERRUPT |
| NM_001184897 | chrX | PHF8 | DUP-INTERRUPT |
